# Evaluation of an Oral Health Management Project in Connection to a Non-Communicable Disease Prevention and Management Project: A Case Study in South Korea

**DOI:** 10.3390/ijerph19095209

**Published:** 2022-04-25

**Authors:** Sang-Hee Yoo, Se-Hwan Jung, Sun-Jung Shin

**Affiliations:** 1 Department of Dental Hygiene, College of Dentistry, Gangneung Wonju National University, Gangneung-si 25457, Korea; ysh3779@nate.com; 2 Research Institute of Oral Science, Gangneung Wonju National University, Gangneung-si 25457, Korea; feeljsh@gwnu.ac.kr; 3 Department of Preventive and Public Health Dentistry, College of Dentistry, Gangneung Wonju National University, Gangneung-si 25457, Korea

**Keywords:** community health planning, community health services, noncommunicable diseases, oral health, people-centered health care, public health

## Abstract

This study aimed to develop, pilot, and evaluate a three-year integrated preventive management project focused on chronic diseases and oral health prevalence. A total of 1148 users of the health care office of the G Public Health Center with dental risk factors were selected for this study and connected to the dental counseling department. Respondents were classified into a group that would receive counseling-type self-education on oral care and a second group that needed dental care. To evaluate the dental care utilization and satisfaction, a telephone survey was conducted with the 263 people. Oral health behavioral changes were analyzed in 97 comparable subjects who responded to both the oral health basic survey and telephone survey. More than 90% of the subjects who visited the dental clinics were positively satisfied with the system for requesting care and with being referred to dental clinics at the public health center or community dental clinics. Measures of oral health perception and of behavior need showed positive changes. This study was effective in inducing positive changes in the oral health management behavior of chronically ill patients and in promoting the use of preventive management-centered dental care.

## 1. Introduction

Non-communicable diseases (NCDs) are widely recognized as a major health-related agenda item in the 21st century; NCDs are responsible for 70% of the global mortality rate and are a major socioeconomic burden [1]. The World Health Organization (WHO) has designated cardiovascular disease, cancer, chronic respiratory diseases, and diabetes as the four major NCDs, and recommends national countermeasures along with the management of risk factors such as smoking, nutrition, and exercise [2]. Periodontal disease, a chronic inflammatory disease, is an oral disease with a high prevalence in the global adult population. Epidemiologic and mechanistic studies on its association with NCDs have been reported. Evidence has suggested that oral microorganisms such as *Streptococcus sanguis* and *Porphyromonas gingivalis* may increase the risk of cardiovascular disease by inducing platelet aggregation and promoting thrombus formation. Chronic gram-negative periodontal infection has been shown to aggravate insulin resistance and affect blood sugar control. However, the intervention of periodontal treatment has proven to be effective in glycemic control by reducing insulin resistance in patients with diabetes. The importance of daily oral care continues to be emphasized as a way to prevent and manage NCDs [3].

Approaches to upstream (macro-level) and downstream (micro-level) interventions in health have emerged as policy measures aiming to solve the fundamental problem of health inequality. For disease prevention and oral health promotion, a strategic intervention approach targeted to upstream factors is now being emphasized over an approach focused on downstream factors [4]. Accompanied by increasing social awareness, upstream approach efforts include establishing policies on smoking cessation, diet, and creating healthy settings [5]. However, for both the individual and the overall population’s health care, it is also important to establish a platform of medical access that targets downstream intervention. The WHO has also suggested the importance of people-centered health care [6]. Previously, NCDs and oral diseases were separated by disease and considered as segmented projects, but the WHO now suggests an action plan for the prevention and promotion of oral health that meets the need for integrated management of non-communicable diseases and oral diseases based on the common risk factor approach (CRFA) [7]. The World Dental Federation has also announced plans for integrated management of oral and chronic diseases based on the CRFA, encouraging health ministries of each country to promote integrated prevention projects [8,9]. However, existent studies in the literature refer only to the association between non-communicable diseases and oral health, or to evaluations of the effectiveness of non-surgical periodontal treatment. Studies on the development and evaluation of integrated programs that can be operated in the community continuously were found to be insufficient. To bridge the known research gap, this study conducted research focused on a downstream medical approach based on the need for integrated management of NCDs and oral health centered on patient care.

In South Korea, the Ministry of Health and Welfare establishes a basic plan for oral health projects in accordance with the Dental Health Act and with the National Health Promotion Comprehensive Plan that presents the national oral health policy. Based on this national plan, local governments establish implementation plans and promote the national project [10]. Health-promotion projects implemented by public health centers were previously designated as centralized (top-down) methods; projects that did not take into account the condition of the community were also promoted. However, since 2013, promotion has changed to a decentralized (bottom-up) method that sets priorities according to the health problems and characteristics of the local community, reflecting the needs of the residents in planning and promoting the project autonomously. In addition to the integrated operation of projects within public health centers, South Korea is considering the common risk factors of chronic diseases, promoting linkage and cooperation projects using local community resources, to provide a target-oriented integrated service rather than a segmented project operation [11].

South Korea’s National Health Plan has included the adult chronic disease-linked gum care project as a core project since 2016. The plan connects patients with chronic diseases whose care is managed at public health centers or local hospitals and clinics to the oral health office of public health centers or local dental hospitals and clinics. The goal is to promote and provide a periodontal management program that is comprised of oral examination (including dental biofilm examination), education and information about oral care methods for chronic diseases, scaling, and other aspects of oral care [12]. However, at the national level, there are still few efforts focused on the development of project guidelines and the operation of regional cooperatives.

Therefore, this study aimed to develop and apply a patient-centered oral health management referral program for people who visit the health care office at the G Health Center in Seoul, South Korea, and to evaluate operational results. The G Health Center was first introduced to the WHO in 2014 as an excellent example of an organization providing community-based services to chronically ill patients [13]. The integrated preventive management program centered on patient care for chronic diseases and oral health that was developed in this study was created in conjunction with primary medical institutions where chronic disease management is conducted systematically. The study’s goals were to improve accessibility at the local level and to create a community network to develop, operate, and evaluate a program that establishes a cooperative system between the dentists’ association in District G and the public health center.

## 2. Methods

### 2.1. Study Subjects and Methods

This study was carried out after obtaining approval from the Research Ethics Committee of Gangneung-Wonju University Dental Hospital (GWNUIRB-2014-8).

In 2014, an oral health management project connected to a community NCDs preventive management project was developed and piloted. The goals were establishing and connecting oral health-related projects in the health care office of the G Health Center in Seoul, South Korea. Based on the results of the pilot project in 2014, a program was conducted in collaboration with local dental clinics in 2015. Prior to the implementation of the program, education and training were conducted for the personnel of the community’s dental clinics to allow them to operate the program; banners to promote the program and educational media for dental clinic waiting rooms were provided. Based on the achievements of the preceding two years, in 2016, the health care office and the oral health office were connected with the public health center, and, with the participation of community dental clinics, the conjoined project was launched. The project participation satisfaction rates and oral health behavior changes were measured and evaluated.

The health care office at the G Health Center conducted basic health questionnaires and tests for blood pressure, fasting blood sugar, triglycerides, HDL-cholesterol, and abdominal circumference. After classifying each patient into a health concern group, a metabolic syndrome group, a drug treatment group, and/or a normal group, the office provided regular services for the prevention and management of chronic diseases (e.g., high blood pressure, diabetes, dyslipidemia, obesity).

**Figure 1:**
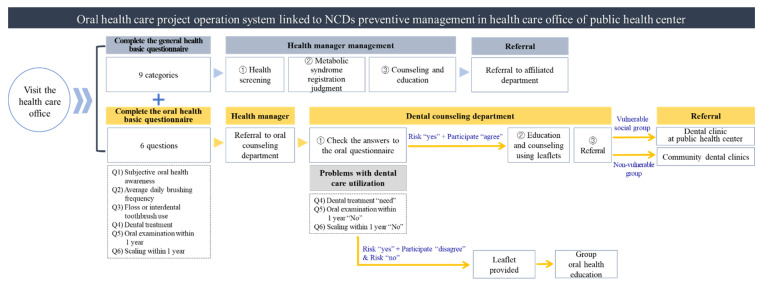
Oral health care and NCD prevention management project system flow.

From 1 December 2015 to 13 December 2016, 1798 patients of the health care office at the G Public Health Center who responded to the oral health basic survey (health concern group: 34.1%; metabolic syndrome group: 18.4%; drug treatment group: 30.8%; normal group: 16.1%) were assessed for the presence of five oral health risk factors (brushing teeth less than three times a day, no use of floss or interdental toothbrush, subjective need for dental treatment, no scaling within one year, and no oral examination within one year). After referring 1148 people who were selected based on the risk factors to the dental counseling department, subjects who had no abnormal dental care use or who did not agree to participate in the program were provided oral health education via leaflets. After receiving oral health education, patients in the vulnerable group were referred to the dental clinic of the public health center; the non-vulnerable participants were referred to community dental clinics after being offered their oral health education (see Figure 1). Of the 353 people who were referred to the public health center and community dental clinics, 263 people (74.5%) agreed to a telephone survey. Those participants were analyzed via a dental care usage and satisfaction evaluation for oral health perception and behavior change. A system malfunction resulting from the establishment of the system in 2015 excluded 166 people from the analysis; data from 97 comparable subjects who responded to both the oral health basic questionnaire and the telephone questionnaire were analyzed.

The dental care usage and satisfaction evaluation questionnaire consisted of four questions that asked for details regarding: if a dental visit occurred, the specifics of the dental treatment provided, about satisfaction with the dental visit, and about satisfaction with the system connected to the public health center. The oral health perception and behavior change questionnaire consisted of four questions that asked about: subjective oral health status, average daily brushing occurrences, floss and interdental toothbrush use, and if dental treatment was needed. Responses were compared with responses from the basic questionnaire.

### 2.2. Statistical Analysis

The collected data were analyzed using IBM SPSS Statistics 23.0 (IBM, Armonk, NY, USA); the significance level for determining statistical significance was set to 0.05. Frequency analysis was performed to evaluate dental care usage and satisfaction. Either a McNemar’s χ^2^ test, a McNemar-Bowker test, or a Wilcoxon signed-rank test was performed to assess oral health perception and behavioral changes. A χ^2^ test or Mann–Whitney U test was performed to compare the oral health perception and behavioral changes between the group with and without dental visits.

## 3. Results

### 3.1. Monitoring the Oral Health Management Project Connected to the NCDs Preventive Management Project

Assessing the 1798 patients from the health care office who responded to the oral health basic survey, 1148 people were found to have oral health risk factors. The 353 people who exhibited dental care utilization risk factors and who agreed to participate in this program received oral health education and counseling through the dental office at the public health center or through a community dental clinic. After conducting a second survey, this time a telephone survey, with 263 of the 353 patients with risk factors, it was found that 92 of the 263 people who agreed to the telephone survey actually visited the dental office of the public health center or a community dental clinic.

**Figure 2:**
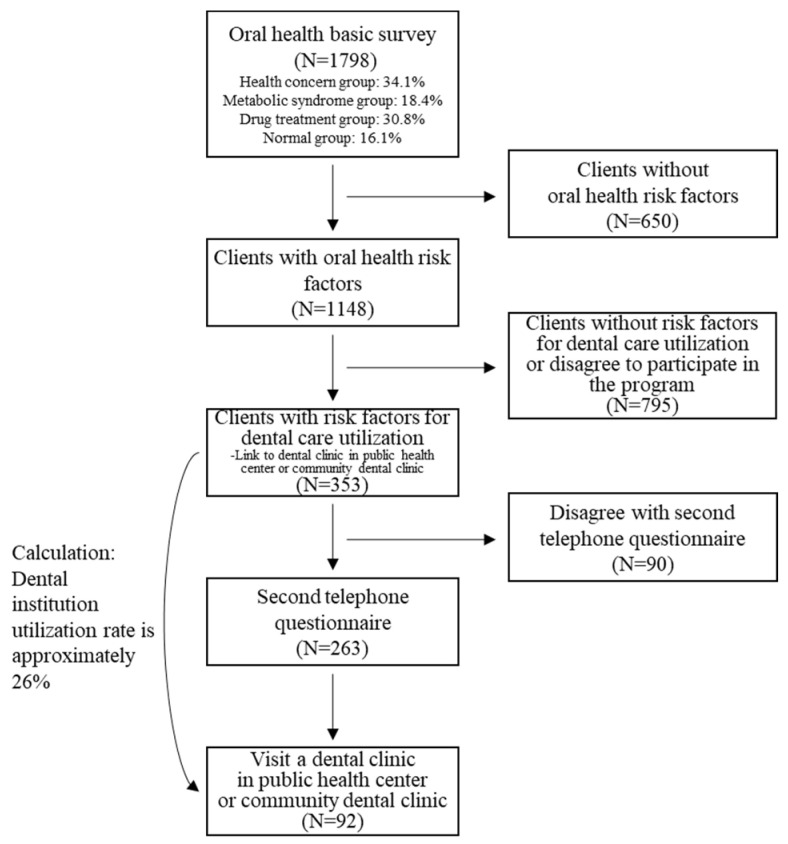
Monitoring flow of the oral health management and NCDs preventive management project.

From the results of selecting subjects with oral health risk factors through the program operation presented in this study, reclassifying subjects with dental care utilization risk factors, and connecting them with dental institutions, it was found that about 8% of the subjects who actually used dental institutions were among the subjects with risk factors for using dental care, and the actual rate of use of dental institutions was calculated to be about 26% (See Figure 2).

### 3.2. Dental Care Usage Status and Satisfaction Survey Connected to the NCDs Preventive Management Project: General Characteristics of Subjects

Survey data about the general characteristics of the subjects demonstrated that more women (74.5%) than men participated in the study; 53.2% of participants were in their sixties. Among participants who had problems accessing and utilizing dental care, 43.0% were connected to the public health center dental office and 57.0% were connected to the community dental clinic. Based on the registration records of the public health center’s health care office, the drug treatment group yielded the highest number of study participants (44.1%, see Table 1).

### 3.3. Dental Care Usage Status and Satisfaction Survey Connected to the NCDs Preventive Management Project: Visiting the Dental Office at the Public Health Center and the Local Community Dental Clinics

After receiving education and counseling related to chronic and oral diseases, 35.2% of the subjects visited the health center’s dental office or a community dental clinic. These subjects were categorized by registration groups formed by the health care office at the public health center as follows: 50% from the normal group, 36.4% from the health concern group and metabolic syndrome group, and 32.2% from the drug treatment group (see Table 2).

### 3.4. Current Dental Care Status in Connection with the NCDs Preventive Management Project: Treatment Provided by Dental Office of the Public Health Center or by a Community Dental Clinic

For subjects connected to the dental office in the public health center, 100% received oral examinations and 95.5% received individualized information and oral health education. For subjects connected to a local dental clinic, 93.6% received oral examinations and 19.1% received individualized information and oral health education (see Table 3).

### 3.5. Satisfaction Survey: Dental Care Usage in Connection with the NCDs Preventive Management Project and the Associated System

Results of the dental care use satisfaction survey indicated that patients connected to the dental office at the public health center showed high satisfaction, with 95.4% reporting that they were “very satisfied-satisfied”; 87.3% of patients connected with the community dental clinic reported that they were “very satisfied-satisfied.” Results of the satisfaction survey focused on the study’s system that provided information about the relationship between chronic diseases and oral health from the public health center, and connected patients to oral health management education through local dental clinics. Results measuring the satisfaction of patients connected to the dental office at the public health center showed that 93.2% of respondents were “very satisfied-satisfied.” Patients who were connected to a community dental clinic reported high system satisfaction, with 100% of the respondents responding, “very satisfied-satisfied” (see Table 4).

### 3.6. Changes in Oral Health Perception and Behavior of Patients in the NCDs Preventive Management Project

Comparing the responses from the first oral health basic questionnaire and the second telephone questionnaire after using the program, in terms of the reported subjective evaluation of oral health awareness, the percentage of respondents who answered “bad” or “very bad” decreased by 12.4% (*p* = 0.136). The average number of brushings per day increased slightly from 2.55 times to 2.74 times (*p* = 0.049); the number of subjects who sought and utilized dental care also increased slightly. The frequency of flossing or interdental brush use increased by 22.7% in the response category “at least once a week” (*p* = 0.001); the use of oral aids was high among the subjects who utilized dental care. In terms of the reported subjective need for dental treatment, the percentage of respondents who answered “very necessary, necessary” decreased from 61.9% to 30.9% (*p* < 0.001); respondents who did not utilize dental care demonstrated a high need for it (see Table 5).

## 4. Discussion

The world is rapidly becoming an aging society and the prevalence of chronic diseases is increasing remarkably.

According to the health status of Korean adults, the prevalence of periodontal disease was 43.5% among patients with diabetes, 41.7% among patients having suffered a stroke, and 37.5% among patients with angina. As compared to normal people, chronic disease patients have a higher risk of periodontal disease, so oral health management of chronic disease patients is emerging as an important task [14]. Accordingly, the National Health Plan of Korea is promoting a chronic disease-linked gum care project for adults as a core project; related programs are being developed that are centered on local government public health centers.

The G Public Health Center in Seoul, South Korea, was selected as the subject of this study. The site not only has installed and operated a health care office within the center, but it also contains a 100-year-old Health Counseling Center with nurses from the community service centers. The health center is easily accessible to residents and provides an integrated management system for chronic diseases such as metabolic syndrome, hypertension, diabetes, dyslipidemia, and obesity. The health center is not only an excellent source of care in Korea, but it was also included within the WHO website in 2014 under the theme of “Community-based prevention of non-communicable disease in Korea.” Since then, the health center has been known as a stellar institution that promotes projects that provide customized health care services for local residents through an established multidisciplinary cooperative system [13].

Considering the high correlation between chronic disease and oral health, this study aimed to create a model that would provide integrated and customized health care services for local residents. The study developed an integrated preventive management project linked with an oral health project that was centered on local residents with chronic diseases and registered with the health care office in the local public health center. The intention was to plan for effective future business operations, evaluate expansion results, and operate as a consultative body with the local dentists’ association.

Among patients with risk factors related to oral health requiring dental care, and who were referred to the dental office at the public health center or to a community dental clinic, 35.2% visited a dental office or clinic and received dental services such as oral examinations and scaling. In accordance with the National Health Insurance system, Korean adults can receive a free oral examination every two years; scaling is covered by insurance once a year. Nationally, the oral examination rate was 39.1% in 2019, which is half of the annual general health examination rate [15]. Although scaling is covered by insurance, its usage is low (16.6% in 2015) [16]. However, approximately one third of the study’s subjects who were referred through the project to dentists visited a dentist and received an oral examination. This rate of occurrence is believed to have led to a positive change for those patients who had not visited the dentist for preventive purposes in the past, encouraging them to visit the dentist for preventive management through the NCDs and oral health management program.

Subjects who were referred to the dental office at the public health center had signed up for medical benefits due to financial difficulties. They primarily received oral examinations and oral health education, considering that these services were found to be helpful in managing oral health and preventing oral health issues. Socioeconomic factors have the greatest influence on the inequality in dental care utilization [17]. The need to develop an oral health project based on health equity has been raised repeatedly. Specifically, the WHO has emphasized the need for interventions based on the relationship between oral health and systemic health [18]. The National Health Plan of Korea (HP2030) reported that health inequality is expected to intensify without appropriate policy interventions from the government [19]. Therefore, the project presented in this study was connected to a dental clinic in a public health center (a primary dental care institution), in consideration of how low-income chronically ill patients seek and utilize dental care. Local public health centers should provide primary care in the community; health projects geared toward underserved populations should be designed and launched so that medical resources can be distributed equitably. As shown in this study, patients who use the public health center program were evenly distributed among vulnerable and general residents. A plan to make available dental clinic-linked services for chronically ill patients with financial difficulties can help lower barriers to dental care access and use. Continued and extended future projects of this kind will influence oral health inequality positively.

The model project presented in this study is an encouraging operational case in which a dentists’ association and a public health center formed a cooperative system to manage the oral health of chronically ill patients. Results of the survey that measured satisfaction with the system that connected patients to a dental office at a public health center or to a community dental clinic yielded a 93.5% positive response. Subjects who visited dental sites reported a satisfaction rate of 91.1%. Subjective oral health awareness, daily average brushing frequency, dental floss or interdental toothbrush use rate, and subjective dental treatment need were all factors that changed positively before and after the project’s implementation. Notably, the frequency of brushing and the use of oral care products were higher among patients who received services connected with a dental institution than those who did not. Study subjects who did not visit a dental site demonstrated high rates of belief that their oral health was unhealthy and that they needed dental treatment.

Through the establishment of community governance, including the Ministry of Health and Welfare, local doctors’ associations, and local clinics, a primary care chronic disease management project is in operation currently in South Korea. Primary medical institutions within the community participate in the project and manage patients with chronic diseases such as hypertension and diabetes [20]. This chronic disease management project establishes an individual care plan for patients with high blood pressure and diabetes, continuously and comprehensively managing treatment, education, and counseling. The program has been shown to be effective through patient compliance and chronic disease control rates [21].

The Ministry of Health and Welfare, local dentists’ associations, local dental clinics, and public health centers should cooperate to establish a similar national system for oral health management for chronically ill patients based on the project model presented in the current study, and continue to promote the project in the future. Beginning in 2007, Australia provided Medicare dental insurance benefits to patients with NCDs as a chronic disease management measure; however, this effort ended in 2012 [22]. Concern about the cost of medical services is a health care system problem that needs to be considered realistically. As the number of people with complex chronic diseases increases, accompanied by population aging, it is important for medical systems to handle complex and chronic diseases smoothly. The WHO emphasizes the necessity of Integrated People-Centered Health Services (IPCHS), which is a paradigm shift from the existing mechanism of the medical system [23]. In addition, ten years after the WHO World Health Assembly established a resolution in 2007 that the oral health sector should integrate with NCDs to promote oral disease prevention and health promotion, the application of the programs to ministries of health around the world was investigated. As a result, it was confirmed that several countries are accepting an integrated approach for oral disease prevention and health promotion, and are implementing intervention programs for diabetes, cardiovascular disease, and CRFA that consider oral health [24]. If an insurance system for patients with NCDs in need of dental care is established in the future, it is expected that oral care programs related to NCDs will be activated at community dental clinics and will contribute to patient-centered medical care.

In this study, an oral health management program connected to NCDs was developed and operated. It is difficult to generalize the effect of this case, but it contributes meaningfully to the literature and to society by presenting a preventive project model for people-centered health care that can manage chronic diseases and oral health in an integrated way. More adult and elderly patients should benefit from integrated management of chronic diseases and oral health, through future expansion of the pilot project operation across regions. In this study, although the effect on the short-term project operation was confirmed, there is a limitation in that the effect of the project in the medium and long-term aspects was not confirmed. It is thought that a systematic system establishment is necessary for project operation. This project should be established as a cooperative system that can be established as a service provided by the local community dental clinics by clarifying the role of the local community dental clinics and participating in monitoring. In terms of the effectiveness of the project, an evaluation that connects with the goals of the oral indices presented in the South Korean National Health Plan (HP 2030) is necessary. To implement the project properly, a qualitative evaluation through continuous monitoring is required.

## 5. Conclusions

The NCDs-oral health management project presented in this study was found to induce positive changes in the oral health management behavior of chronically ill patients. The model project was effective in promoting the use of preventive management-centered dental care. Future expansion of the project operation across regions and continuous monitoring through the establishment of community governance are recommended.

## Figures and Tables

**Table 1:** General characteristics of subjects.

Division		N	%
Total		263	100.0
Gender	Male	67	25.5
	Female	196	74.5
Age	20–29	3	1.1
	30–39	7	2.7
	40–49	19	7.2
	50–59	66	25.1
	60–69	140	53.2
	≥70	28	10.6
Referral classification	Dental clinic in the public health center	113	43.0
	Community dental clinic	150	57.0
Health care office registration group *	Health concern group	78	29.7
	Metabolic syndrome group	55	20.9
	Drug treatment group	116	44.1
	Normal group	14	5.3

* Health concern group: people with 1 or 2 risk factors out of 5 (blood pressure, fasting blood sugar, triglycerides, HDL-cholesterol, and abdominal circumference). Metabolic syndrome group: people with 3 to 5 risk factors out of 5. Drug treatment group: people currently taking medication regardless of the number of risk factors. Normal group: people with 0 risk factors out of 5.

**Table 2:** Visits to the dental office at the public health center or the local community dental clinics.

Division	Total	Yes	No
Total	261 (100.0)	92 (35.2)	169 (64.8)
Health care office registration group			
Health concern group	77 (100.0)	28 (36.4)	49 (63.6)
Metabolic syndrome group	55 (100.0)	20 (36.4)	35 (63.6)
Drug treatment group	115 (100.0)	37 (32.2)	78 (67.8)
Normal group	14 (100.0)	7 (50.0)	7 (50.0)

Values are presented as n (%). Analysis was performed for all participants except for those who did not respond.

**Table 3:** Treatment provided by public health center dental office or community dental clinic.

Division	Clients Linked to the Public Health Center Dental Office					Clients Linked to Community Dental Clinics				
	Total	Health Concern Group	Metabolic Syndrome Group	Drug Treatment Group	Normal Group	Total	Health Concern Group	Metabolic Syndrome Group	Drug Treatment Group	Normal Group
Total	44 (100.0)	15 (100.0)	9 (100.0)	18 (100.0)	2 (100.0)	47 (100.00)	12 (100.0)	11 (100.0)	19 (100.0)	5 (100.0)
Oral examination										
Yes	44 (100.0)	15 (100.0)	9 (100.0)	18 (100.0)	2 (100.0)	44 (93.6)	10 (83.3)	11 (100.0)	18 (94.7)	5 (100.0)
No	0 (0.0)	0 (0.0)	0 (0.0)	0 (0.0)	0 (0.0)	3 (6.4)	2 (16.7)	0 (0.0)	1 (5.3)	0 (0.0)
Information provision/oral health education (related to chronic disease and oral health)										
Yes	42 (95.5)	13 (86.7)	9 (100.0)	18 (100.0)	2 (100.0)	9 (19.1)	1 (8.3)	1 (9.1)	6 (31.6)	1 (20.0)
No	2 (4.5)	2 (13.3)	0 (0.0)	0 (0.0)	0 (0.0)	38 (80.9)	11 (91.7)	10 (90.9)	13 (68.4)	4 (80.0)

Values are presented as n (%). Analysis was performed for all participants except for those who did not respond.

**Table 4:** Satisfaction with dental care through the NCDs preventive management project.

Division	Satisfaction with Dental Care			Satisfaction with the Referral System		
	Total	Clients Linked to Dental Clinic in Public Health Center	Clients Linked to Community Dental Clinic	Total	Clients Linked to Dental Clinic in Public Health Center	Clients Linked to Community Dental Clinic
Total	90 (100.0)	43 (100.0)	47 (100.0)	259 (100.0)	44 (100.0)	46 (100.0)
Very satisfied	37 (41.1)	27 (62.8)	10 (21.3)	154 (59.5)	31 (70.5)	29 (63.0)
Satisfied	45 (50.0)	14 (32.6)	31 (66.0)	88 (34.0)	10 (22.7)	17 (37.0)
Normal	6 (6.7)	2 (4.7)	4 (8.5)	16 (6.2)	3 (6.8)	0 (0.0)
Not satisfied	0 (0.0)	0 (0.0)	0 (0.0)	1 (0.4)	0 (0.0)	0 (0.0)
Not satisfied at all	2 (2.2)	0 (0.0)	2 (4.3)	0 (0.0)	0 (0.0)	0 (0.0)

Values are presented as n (%). Analysis was performed for all participants except for those who did not respond.

**Table 5:** Oral health awareness, average daily brushing frequency, floss and interdental toothbrush use, and need for dental treatment.

Division	First Oral Health Basic Questionnaire	Second Telephone Questionnaire				
		Total	*p* *	Visited Dental Clinic	No Visit to Dental Clinic	*p* **
Total	97 (100.0)	97 (100.0)		32 (100.0)	65 (100.0)	
Subjective oral health awareness						
Very good, good	21 (21.6)	25 (25.8)	0.136	10 (31.3)	15 (23.1)	0.609
Normal	38 (39.2)	46 (47.4)		15 (46.9)	31 (47.7)	
Bad, very bad	38 (39.2)	26 (26.8)		7 (21.9)	19 (29.2)	
Average daily brushing frequency	2.55 ± 0.71	2.74 ± 0.85	0.049	2.97 ± 0.86	2.63 ± 0.82	0.064
Floss and interdental toothbrush use						
Not used	48 (49.5)	26 (26.8)	0.001	5 (15.6)	21 (32.3)	0.081
At least once a week	49 (50.5)	71 (73.2)		27 (84.4)	44 (67.7)	
Subjective need for dental treatment						
Very necessary, necessary	60 (61.9)	30 (30.9)	<0.001	6 (18.8)	24 (36.9)	0.609
Unnecessary, absolutely unnecessary	37 (38.1)	67 (69.1)		26 (81.3)	41 (63.1)	

Values are presented as n (%) or mean ± standard deviation. * *p*-values were calculated either by McNemar-Bowker test, McNemar chi-square test, or Wilcoxon signed-rank test by comparing the first and the second questionnaires’ results. ** *p*-values were calculated by chi-square test or Mann–Whitney U test according to the presence or absence of a dental visit.

## Data Availability

Not applicable.
